# Addressing health inequities in Ontario, Canada: what solutions do the public support?

**DOI:** 10.1186/s12889-016-3932-x

**Published:** 2017-01-05

**Authors:** Maritt Kirst, Ketan Shankardass, Sonica Singhal, Aisha Lofters, Carles Muntaner, Carlos Quiñonez

**Affiliations:** 1 Department of Psychology, Wilfrid Laurier University, 75 University Ave. West, Waterloo, ON N2L 3C5 Canada; 2 Dalla Lana School of Public Health, University of Toronto, Toronto, Canada; 3 Centre for Urban Health Solutions, Li Ka Shing Knowledge Institute, St. Michael’s Hospital, Toronto, Canada; 4 Department of Health Sciences, Wilfrid Laurier University, Waterloo, Canada; 5 Faculty of Dentistry, University of Toronto, Toronto, Canada; 6 Public Health Ontario, Toronto, Canada; 7 Department of Family and Community Medicine, University of Toronto, Toronto, Canada; 8 Bloomberg Faculty of Nursing, University of Toronto, Toronto, Canada; 9 Program in Mind-Society Interaction, Korea University, Seoul, South Korea

## Abstract

**Background:**

As public opinion is an important part of the health equity policy agenda, it is important to assess public opinion around potential policy interventions to address health inequities. We report on public opinion in Ontario about health equity interventions that address the social determinants of health. We also examine Ontarians’ support and predictors for targeted health equity interventions versus universal interventions.

**Methods:**

We surveyed 2,006 adult Ontarians through a telephone survey using random digit dialing. Descriptive statistics assessed Ontarians’ support for various health equity solutions, and a multinomial logistic regression model was built to examine predictors of this support across specific targeted and broader health equity interventions focused on nutrition, welfare, and housing.

**Results:**

There appears to be mixed opinions among Ontarians regarding the importance of addressing health inequities and related solutions. Nevertheless, Ontarians were willing to support a wide range of interventions to address health inequities. The three most supported interventions were more subsidized nutritious food for children (89%), encouraging more volunteers in the community (89%), and more healthcare treatment programs (85%). Respondents who attributed health inequities to the plight of the poor were generally more likely to support both targeted *and* broader health equity interventions, than neither type. Political affiliation was a strong predictor of support with expected patterns, with left-leaning voters more likely to support both targeted and broader health equity interventions, and right-leaning voters less likely to support both types of interventions.

**Conclusions:**

Findings indicate that the Ontario public is more supportive of targeted health equity interventions, but that attributions of inequities and political affiliation are important predictors of support. The Ontario public may be accepting of messaging around health inequities and the social determinants of health depending on how the message is framed (e.g., plight of the poor vs. privilege of the rich). These findings may be instructive for advocates looking to raise awareness of health inequities.

## Background

Socio-economic position is one of the most important determinants of health inequities within societies. Health inequities are differences in health between specific population groups that are systematic, avoidable, unfair and unjust [1–4]. Health inequities systematically place individuals who are already socially disadvantaged, in terms of income, gender, race and/or ethnicity, at further disadvantage related to health [1, 5]. Globally, there is momentum to investigate and act upon health inequities by strengthening the social determinants of health, including early childhood development, adequate income, fair income distribution, high educational attainment, non-precarious employment, safe working conditions, food security, and affordable housing. Due to the power of government at all jurisdictional levels (i.e., local, regional, provincial, national) to enact policies that facilitate structural improvements to the social determinants of health, strengthening political will or motivation to act, is a critical concern for addressing health inequities. In Canada’s most populous province of Ontario, while there has been some recent action in creating more equitable access to the healthcare system [6], there has been little action to address other social determinants of health. Such policy inaction could be due to a number of factors, including competing political and social agendas or the status of economic development, but may also be due to a lack of political will (i.e., insufficient buy-in from elected politicians and other political elites) to tackle health inequities [7, 8].

Research has shown that in democratic countries, public opinion can influence political buy-in and public policy outcomes especially in the areas of social welfare and poverty policy [9, 10]; the more salient an issue is to the public, the stronger the relationship to the formulation of the policy agenda [9, 11, 12]. However, only a few studies exist that have examined public opinion on the types of solutions that could address health and social inequities, and findings are mixed. In one American study on public support for welfare policies, support for progressive policies (e.g., extended child care and health benefits, cash benefits that are adjusted for cost of living increases) was predicted by social/structural attributions for poverty, whereas support for restrictive welfare policies (e.g., fingerprinting welfare recipients, “family cap,” reducing benefits if children do not attend school regularly) was predicted by individualistic attributions emphasizing individual responsibility for poverty and wealth [13]. Another US study found a strong relationship between beliefs that health inequalities were unfair and support for a government-funded health insurance plan [14]. An American study examining how public perceptions of the poor affect support for social policies related to poverty found that liberal assistance policies were more likely to be recommended when the target group was perceived to be more deserving (i.e., the physically handicapped); however, conservative policies were supported when the target group was perceived to be undeserving (i.e., able-bodied men) [15].

In the Canadian context, a study of public support for government spending in impoverished areas in the province of Alberta found that the public showed the greatest support for child care programs and the least support for increased welfare allowance [9]. A similar study in the province of Saskatchewan found that participants were most willing to support earning supplements for welfare recipients and strengthening early intervention programs for infants. Less support was observed for subsidized food and recreation, despite the near-unanimous opinion that these are major determinants of health [2]. Another recent study in Saskatchewan identified patterns in support for health equity policies, finding that 37% were in a selective agreement category, whereby only a select number of policies were supported, and the remaining 63% had high agreement across a range of policy options. The select agreement group showed lower policy support for a guaranteed annual income, increased welfare for adults and parents with children, and lower tuition fees for post-secondary students, than did the high agreement group [16]. While these studies have all examined public support for health equity interventions, few studies in the Canadian context have explored public attributions of health inequities and how they are related to support for health equity interventions. As public opinion can be an important part of determining the health equity policy agenda, it is of interest to monitor the state of public knowledge and opinion around potential policy interventions to address health inequities. Given the above-mentioned findings in the existing literature, it is also important to gauge the extent to which the public will support different types of interventions, i.e. whether they are universal (i.e., applicable to all citizens), or targeted to specific subpopulations, as well as reasons for such support. This information is useful for the development of messaging to improve public awareness and understanding on this issue for the purpose of strengthening political will for health equity interventions. In this paper, we report on public opinion in Ontario about health equity policy interventions that address the social determinants of health. We also engage a debate in the health equity literature that considers whether policy interventions to reduce health inequities should be universal or targeted to the socially disadvantaged [3, 7, 17, 18], by exploring predictors of Ontarians’ support for targeted health equity interventions versus interventions that affect the broader population. As part of these analyses, we examine the relationship between how the public attribute health inequities and support for targeted vs. broader health equity interventions.

## Methods

### Data collection

We surveyed 2,006 Ontarians aged 18 years and over through a telephone interview using random digit dialing. A market-based research firm (Opinion Search) administered the survey. Landline telephone numbers were randomly sampled, but quotas based on the Ontario population for sex, age, and geography (urban/rural) were imposed. 69,906 numbers were called and of these, there were 56,528 eligible calls (excluding numbers that were not in service, fax machines, or invalid). After exclusions for busy signals, answering machines, no answer, language barrier, ill or incapable participants, and eligible persons not available, a total of 33,530 individuals were asked to participate. Only one person was chosen from each household. Overall, a response rate of 5.49% was achieved, with 9.24% of persons asked to complete the survey doing so.

The survey asked a series of questions related to three thematic areas: 1) awareness of health inequities; 2) explanations or attributions of health inequities; and 3) opinions about possible solutions to health inequities. Findings on the first two thematic areas have been reported in previous publications [6, 19]. The present analyses reflect responses to questions in the third thematic area on possible solutions to health inequities. The questions on possible solutions asked participants to respond, according to a 5-item Likert scale of agreement, to sets of statements in the following areas: a) the importance of addressing health inequities in Ontario; b) fairness in health status in Ontario; c) possible intervention approaches to address health inequities in the province; and d) support for specific intervention types. Individuals were asked if they would participate in the survey, and willingness to complete the survey was taken as verbal consent. Ethics approval for the study, including these consent procedures, was received from the University of Toronto Research Ethics Board.

### Data analysis

We calculated the proportion of responses for each of the sets of survey items. We then classified responses as binary measures indicating some level of agreement (strongly agree and agree), versus some level of disagreement or a neutral response (strongly disagree, disagree and neither agree nor disagree), as we were most interested with agreement with the statements. To engage the debate as to whether social policy interventions to reduce health inequities should be targeted or universal [17], we grouped interventions based on whether they were targeted (i.e., those targeted to specific subpopulations, in this case families with children), or so-called universal or broader interventions affecting larger groups by comparison (e.g., those benefitting all citizens), as well as by social determinants of health. We then calculated response proportions by these types. Descriptive statistics were calculated on each of these groupings.

In multivariate analyses, we examined the relationship between three categories of attributions of health inequities (identified in Lofters et al., 2014) and support for a subset of six interventions. The categories of attributions of health inequities were defined based on agreement with one of three explanations of health inequities: ‘blaming the poor’, an explanation that blames unhealthy behaviours of lower income groups for health inequities; ‘plight of the poor’, an explanation that attributes health inequities to the socio-economic disadvantage of lower income groups; and ‘privilege of the rich’, an explanation that attributes health inequities to the relative wealth and health of the rich [6]. Three of the six interventions examined in these analyses were targeted interventions for families with children, and the other three were equivalent but broader interventions. These interventions included housing interventions (e.g., targeted: quality housing for parents with children; broader: quality housing), welfare interventions (e.g., targeted: increasing welfare amounts to above poverty level for parents with children; broader: increasing welfare amounts to above poverty level), and nutrition interventions (e.g., targeted: more subsidized nutritious food for children; broader: more subsidized nutritious food) (see Table 2). Housing, welfare and nutrition interventions were chosen for this set of analyses because separate questions on support for targeted and broader versions of these particular types of interventions were asked in the survey. Respondents were classified as supportive of targeted, broader, both or neither (reference category) for each set of interventions.

Bivariate analysis was first conducted to assess relationships of each attribution with type of intervention. Following that, a base multinomial logistic regression model was prepared for each set of interventions (housing, welfare and nutrition) to include all three types of attributions as predictors. Adjusted models were then run for all possible predictors including demographic variables and political affiliation. Demographic variables included: sex, age group (18–34 years, 35–54 years, 55+ years), area of residence (urban vs. rural), immigration status (immigrated more than 10 years ago, immigrated 10 years ago or less, Canadian-born), visible minority status (did not report Canadian, American or European ethnic ancestry), total annual household income (< $20,000, $20,000–, $40,000, $40,000 –$60,000, $60,000 –,$80,000, $80,000 –,$100,000, $100,000+), highest attained education (high school diploma or lower versus higher than high school), and whether participants were employed at the time of the survey. Political affiliation was assessed in response to the question, “If the election were being held today, do you think you would vote for the Progressive Conservative, Liberal, New Democratic Party (NDP), Green, or some other candidate?” The former three parties are the parties currently represented in the Ontario legislative assembly, and can generally be defined as right to left wing, respectively. Significant predictors in the bivariate analysis, which changed the parameter estimate of the base models by 10%, were tested in the final models by using the forward-stepwise method. For all analyses, data were weighted to replicate provincial population distributions, by age and sex, according to 2006 Canadian Census data. IBM Statistical Package for the Social Sciences (SPSS) for Windows software (version 22) was used for all analyses.

## Results

Survey respondent characteristics have been reported elsewhere [6, 19]. Briefly, 52% of the sample were female, 40% were aged in between 34 and 54 and 32% were aged 55 and over. Seventy-six percent were born in Canada, and 17% were visible minorities. Study participants were generally representative of the Ontario population based on the 2006 Census (the last comprehensive census with accessible data). Table 1 illustrates the percentage of survey respondents who agreed with statements about the importance of addressing health inequities in Ontario. When asked to rate the extent to which health inequities are a problem in the province of Ontario, responses averaged a 6 on a scale of 1 to 10. Eighty-three percent of respondents agreed that it is important for the government to find ways of narrowing differences in health between the rich and poor. However, 64% agreed that people should take responsibility for their own health and not expect the government to address this. With respect to fairness and health status, 98% of respondents felt that everyone in Ontario should have the same opportunity to live a long and healthy life. Yet, only 47% agreed that everyone in Ontario actually has this opportunity, and 58% agreed that Ontario society needs major changes in order to make things more equitable among citizens.

**Table 1:** Importance of addressing health inequities – percent agreement (*N* = 2,006)

	% (#)
It is important for governments to find ways of narrowing differences in health between the rich and the poor	83 (1661)
People should take responsibility for their own health and not expect the government to do it for them	64 (1286)
Government should work to close the health gap between the rich and poor even if it means raising taxes	48 (957)
Government should work to close the health gap between the rich and the poor even if it means shifting resources away from the better off to the less well off	65 (1306)
On a scale from 0 to 10, where 0 means an issue is not a problem at all and 10 means it is a very big problem, how big a problem do you think the health gap between the rich and poor is in Ontario – Mean (SD) (*N* = 1940)	5.88 (2.20)
If the government were willing and able to spend whatever was necessary, the government *could* eliminate the health gap between the rich and the poor	54 (1080)
Everyone in Ontario should have the same opportunity to live a long and healthy life - Yes	98 (1960)
Everyone in Ontario does have the same opportunity to live a long and healthy life - Yes	47 (942)
Do you think Ontario society needs major changes in order to make things more equal among its citizens? - Yes	58 (1153)

Just under half of respondents (48%) agreed that the government should address health inequities by raising taxes, and 65% agreed that the government should address health inequities through redistributive processes, whereby resources are shifted away from the wealthy to support the poor. Fifty-four percent of respondents agreed that the government, if willing, could eliminate health inequities in Ontario.

With respect to support for specific interventions to address health inequities in Ontario (Table 2), more than 80% of respondents would support strengthening early intervention programs for infants, providing subsidized trades training for adults, providing more healthcare treatment programs, and providing more health prevention programs. Furthermore, more than 80% would support providing more health services in schools, more subsidized nutritious food for children, and encouraging more volunteers in the community. Overall, when regrouping these interventions by social determinants of health (Table 3), we observed the strongest support for health services interventions and nutrition interventions. When examining support for interventions according to whether they are targeted to subpopulations or broader (Table 4), we noted greater support for targeted interventions that benefit age-defined subpopulations like children and seniors (78%), than broader interventions that can benefit everyone, such as more subsidized transit, employment equity programs, and providing more healthcare treatment programs, or that can benefit everyone who is eligible, such as increasing welfare amounts (71%).

**Table 2:** If health does differ between the rich and the poor, what would you support to address this difference? (*N* = 2,006)

	% (#)
Employment and income interventions	
Increasing pension amounts for seniors	80 (1607)
Creating work-earning supplements for welfare recipients	72 (1451)
Increasing welfare amounts to above poverty level for parents with children	71 (1442)
Employment equity programs	70 (1405)
Increasing minimum wage	69 (1396)
Increasing welfare amounts to above poverty level	61 (1226)
Increasing union membership for workers	33 (661)
Education and training interventions	
Providing more subsidized trades training for adults	83 (1675)
Strengthening early intervention programs for infants	82 (1653)
Increasing funding for education	80 (1611)
Creating more after-school or after-work literacy programs	80 (1610)
Creating more subsidized daycares and pre-schools	71 (1433)
Health services interventions	
Providing more health care treatment programs	85 (1700)
Providing more health prevention programs	84 (1696)
Providing more health services in schools	83 (1669)
Housing, transit and recreation interventions	
More subsidized quality housing for parents with children	77 (1536)
More subsidized quality housing	71 (1421)
More subsidized transit	64 (1287)
More subsidized recreation	66 (1314)
Nutrition interventions	
More subsidized nutritious food for children	89 (1788)
More subsidized nutritious food	80 (1594)
Social capital/community engagement interventions	
Encouraging more volunteers in the community	89 (1791)
Creating more community groups and social support networks	71 (1415)
Giving those that are less well-off more ability to influence government decisions	62 (1238)

**Table 3:** Average percentage of respondents who support interventions grouped by the social determinant of health that they address (*N* = 2,006)

	Percent
Support for health services interventions	84
Support for nutrition interventions	84
Support for education and training interventions	79
Support for social capital/community engagement interventions	74
Support for housing, transit and recreation interventions	70
Support for employment and income interventions	65

**Table 4:** Average percentage of respondents who support targeted vs. broader equity interventions (*N* = 2,006)

	Percent
Support for targeted interventions (e.g., interventions targeted towards subpopulations like children, seniors (9))	78
Support for broader interventions (e.g., interventions that benefit everyone such as more subsidized transit, employment equity programs, provide more healthcare treatment programs, etc. (15))	71

Table 5 presents adjusted analyses examining the relationship between attributions of health inequities and support for housing, welfare and nutrition interventions. In the area of housing interventions, people who attributed health inequities to the plight of the poor were more likely to support both targeted housing interventions for families with children and broader housing interventions, than neither intervention (OR: 2.13; CI 95%: 1.64-2.76; *p* < 0.001). Those who vote NDP were 3 times more likely to support both targeted and broader housing interventions, than neither intervention (OR: 3.17; CI 95%: 1.78–5.66; *p* < 0.001). Those who vote for the Conservative party were less likely to support both targeted and broader housing interventions (0.59: CI 95%: 0.45–0.77; *p* < 0.001), and those who were employed were less likely to support targeted housing interventions (OR: 0.34; CI 95%: 0.14–0.84; *p* = 0.020) as well as both types of interventions (OR: 0.30; CI 95%: 0.14–0.64; *p* = 0.002). Foreign-born respondents were more likely to support broader housing interventions than neither (OR: 1.88; CI 95%: 1.16–3.04; *p* = 0.010).

**Table 5:** Multinomial logistic regression analyses (adjusted) of the relationship between health inequity attributions and support for housing, welfare and nutrition interventions to address health inequities

	Housing interventions			Welfare interventions			Nutrition interventions		
Predictor Variable	Targeted only^a^ OR (CI 95%)	Broader only OR (CI 95%)	Both OR (CI 95%)	Targeted only OR (CI 95%)	Broader only OR (CI 95%)	Both OR (CI 95%)	Targeted only OR (CI 95%)	Broader only OR (CI 95%)	Both OR (CI 95%)
Blamers (ref: group: those who did not agree with statements blaming the poor)	0.90 (0.63–1.29)	0.89 (0.56–1.42)	0.79 (0.61–1.02)	1.06 (0.78–1.44)	0.88 (0.54–1.42)	0.64 (0.51–0.81)***	1.57 (1.01–2.43)*	2.10 (1.07–4.12)*	1.40 (0.97–2.01)
Plight of the poor (ref group: those who did not agree with plight of the poor)	1.03 (0.72–1.48)	1.39 (0.87–2.20)	2.13 (1.64–2.76)***	1.09 (0.80–1.48)	1.67 (1.03–2.71)*	1.78 (1.41–2.25)***	1.42 (0.91–2.21)	1.56 (0.80–3.03)	2.04 (1.41–2.95)***
Privilege of the rich (ref. group: those who did not agree with privilege of the rich)	1.25 (0.79–2.00)	0.60 (0.36–1.02)	1.36 (0.97–1.89)	1.14 (0.77–1.67)	0.75 (0.42–1.31)	1.74 (1.28–2.35)***	--	--	--
Conservatives (ref. group: Non-Conservatives)	0.88 (0.61–1.28)	0.73 (0.45–1.21)	0.59 (0.45–0.77)***	1.71 (0.98–2.99)	1.75 (0.74–4.11)	0.64 (0.50–0.82)***	0.76 (0.49–1.18)	0.36 (0.16–0.78) **	0.52 (0.36–0.75)***
NDPs (ref. group: non-NDP)	1.83 (0.86–3.93)	1.46 (0.55–3.91)	3.17 (1.78–5.66)***	0.73 (0.52–1.01)	1.00 (0.60–1.67)	2.21 (1.41–3.46)***	--	--	--
Males (ref. group: Female)	0.98 (0.69–1.39)	0.96 (0.62–1.50)	0.65 (0.51–0.84)***	--	--	--	0.41 (0.19–0.89)*	0.62 (0.21–1.88)	0.33 (0.16–0.67)**
Employed (ref. group: unemployed)	0.34 (0.14–0.84)**	0.46 (0.15–1.42)	0.30 (0.14–0.64)**	--	--	--	--	--	--
Foreign born (ref. group: Canadian-born)	1.46 (0.98–2.17)	1.88) (1.16–3.04)**	1.10 (0.82–1.48)	0.83 (0.58–1.18)	1.75 (1.07–2.88)*	1.08 (0.83–1.40)	--	--	--
Majority (ref. group: minority)	--	--	--	--	--	--	0.74 (0.48–1.14)	1.02 (0.53–1.99)	0.59 (0.41–0.84)**

*p < 0.05

**p < 0.01

***p < 0.001

^a^Reference category is ‘neither intervention’ in all models

With respect to predictors of support for welfare interventions, people who blamed the poor for health inequities were less likely to support both targeted interventions (for families with children) and broader interventions (OR: 0.64; CI 95%: 0.51–0.81; *p* < 0.001), than neither intervention. Those who attributed health inequities to the plight of the poor were more likely to support broader interventions (OR: 1.67; CI 95%: 1.03–2.71; *p* = 0.037) and to support both types (OR: 1.78; CI 95%: 1.41–2.25; *p* < 0.001). Those who attributed inequities to the privilege of the rich were also more likely to support both interventions (OR: 1.74; CI 95%: 1.28–2.35; *p* < 0.001), as were those who vote for the NDP party (OR: 2.21; CI 95%: 1.41–3.46; *p* = 0.001). Foreign-born respondents were more likely to support broader welfare interventions (OR: 1.75; CI 95%: 1.07–2.88; *p* = 0.026).

In the area of nutrition interventions, respondents who blamed the poor for health inequities were more likely to support targeted interventions (i.e., more subsidized nutritious food for children) (OR: 1.57; CI 95%: 1.01–2.43; *p* = 0.044) and broader nutrition interventions (i.e., more subsidized nutritious food) (OR: 2.10; CI 95%: 1.07–4.12; *p* = 0.031), than neither intervention. Those who attribute health inequities to the plight of the poor were more likely to support both targeted and broader nutrition interventions (OR: 2.04; CI 95%: 1.41–2.95; *p* < 0.001). Conservative voters were less likely to support broader nutrition interventions, as well as both targeted and broader interventions of this type (OR: 0.36; CI 95%: 0.16–0.78; *p* = 0.010 and OR 0.52; CI 95%: 0.36–0.75; *p* < 0.001 respectively). Males were less likely to support targeted as well as both targeted and broader nutrition interventions (OR: 0.41; CI 95% 0.19–0.89; *p* = 0.025 and OR: 0.33; CI 95%: 0.16–0.67; *p* = 0.002 respectively). Those who did not identify as members of a minority group were less likely to support both targeted and broader nutrition interventions (OR: 0.59; CI 95%: 0.41–0.84; *p* = 0.004).

## Discussion

Our results have shown mixed opinions among Ontarians regarding the importance of addressing health inequities and related solutions. Almost all respondents (98%) felt that everyone in Ontario should have the same opportunity for a long and healthy life. Less than half (47%) felt that everyone does have the same opportunity to live a long and healthy life. Yet, only 58% felt that Ontario society needs major changes to make things more equitable. Eighty-three percent of respondents felt the government should take action on health inequities, yet, 64% felt it is not the government’s responsibility to act on this issue. Approximately half also support raising taxes as a solution to addressing health inequities. This sample of Ontarians was willing to support a wide range of specific interventions to address health inequities. Among the most supported interventions were more subsidized nutritious food for children (89%), and more healthcare treatment programs (85%). These findings may reflect a general comfort among the public with prominent discourse around the necessity of these kinds of interventions based on deservingness [9], and discontent with the healthcare care system [20]. Popular discourses, for example media discourse, can be important for the formation of public opinion [21], which in turn can influence health policy.

Overall, we found greater support for interventions targeted to specific subpopulations, namely children, than those applicable to a broader population. This is consistent with research that has found that the public is more likely to support interventions for populations deemed deserving, such as vulnerable children who are commonly viewed as innocent and dependent [2, 8, 9]. Yet, earlier findings from this telephone survey highlighted a lack of understanding about the importance of childhood experiences as a determinant of health inequalities for adult Ontarians [6]. This suggests that strengthening the welfare state for all may require health equity advocates to popularize the concept of the life course perspective by emphasizing the importance of resources and supports in early childhood to produce healthy children, and prevent ill health and disadvantage in adulthood.

When types of interventions were grouped by social determinants of health, we found that the highest support was for health services and nutrition interventions. Public support for these particular interventions is likely due to a more obvious connection with health, whereas the public may not see the connection between the other types of interventions and health (e.g., education programs, housing and transit interventions).

With respect to the relationship between attributions of health inequities and Ontarians’ support for housing, welfare and nutrition interventions that were either broad or targeted to families with children, we found that respondents who attributed health inequities to the plight of the poor were generally more likely to support both targeted and broader health equity interventions than neither type. Political affiliation appears to be an important predictor of support with expected patterns, with left-leaning NDP voters more likely to support both targeted and broader health equity interventions, and right-leaning Conservative voters less likely to support both types of interventions.

Our findings are consistent with those of similar studies in the Canadian context that have examined public support for health equity interventions. A recent Toronto Public Health report outlined the magnitude of health inequity in the city, and advocated that governments need to be doing more to act on the social determinants of health to improve equity [22]. Similar to findings in Alberta and Saskatchewan [2, 9], Ontarians showed low support for increased taxation, and greater support for programs targeted towards children. However, unlike findings in Saskatchewan [2], Ontarians in our sample showed equally strong support for nutrition programs. Our findings are also consistent with other research on attribution of health inequities in that they confirm that patterns in this outcome are highly related to the types of interventions that individuals will support [13–15, 23]. As per this research, attributions of social conditions to internal or external factors are influenced by personal experience and socialization to norms and values from the groups with which individuals identify (e.g., socio-economic status, political affiliation) and related perceptions of deservingness [23–25]. Thus, the observed patterns with respect to attributions and intervention support may be due to respondents’ personal experience with the social determinants of health, and/or the societal values that they hold related to political affiliation [25].

Collectively, findings from this and our earlier studies [6, 19] suggest that the Ontario public may be accepting of messaging around health inequities and the social determinants depending on how the message is framed (e.g., based on blaming the poor, the plight of the poor, or the privilege of the rich). In turn, all of this has implications for how Ontarians currently view health equity-related policy interventions. Public support for equity-related interventions might be maximized if advocates and policymakers can convince the public that inequities exist because of the “plight of the poor” and that deserving groups subject to structural forces beyond their control will benefit through these interventions.

Limitations to the study have been documented elsewhere [6, 19]. Briefly, they include limitations related to telephone survey sampling and the exclusion of cellphones, as well as a low response rate. However, our sample is arguably representative, reflected in similar annual household income to the Ontario adult population. To reduce the likelihood of nonresponse error, quota sampling for sex, age and regional representation was used and the data were weighted by age and sex as per the 2006 Canadian Census. Yet the omission of non-English-speaking participants from the survey limits the representativeness of the sample and thus generalizability of findings. Further, for some questions, participants may have interpreted their meaning differently. Furthermore, social desirability bias may have prevented respondents from agreeing with statements that seemed to lay blame on individuals for health inequities, and our constructs of blame, plight and privilege were not tested for their validity. However, these constructs are supported by the health promotion and health inequities literature [23, 26, 27]. An interesting area for future research would involve an experimental study to test the specific impact of message framing by blame, plight and privilege constructs on public support for health equity interventions.

## Conclusions

While both targeted and broader, universal interventions are being implemented worldwide to address health inequities [7], there is little evidence on public support of these interventions, especially in the Canadian context. Our study findings underscore the need to raise greater awareness of health inequities, and develop appropriate messages to promote public support for health equity interventions [25], in so far as public opinion is influential for policy-making. In this regard, more messaging that frames health inequities as due to the plight of the poor or the privilege of the rich could translate into support for the comprehensive range of targeted *and* broader health equity interventions that are needed to tackle this issue. These findings could thus be instructive for advocates looking to raise awareness of health inequities.
